# Endoscopic ultrasound guided bypass of malignant afferent limb obstruction with an electrocautery-enhanced lumen‑apposing metal stent

**DOI:** 10.1055/a-2900-8823

**Published:** 2026-07-07

**Authors:** Karthic Drishna Perumal, Erica Park, Mitchell L. Ramsey, Susan Tsai, Somashekar G. Krishna, Georgios I. Papachristou, Jordan Burlen

**Affiliations:** 1 Internal Medicine 24596 Mount Carmel Health SystemGrove CityOhioUnited States; 2 Division of Gastroenterology, Hepatology and NutritionThe Ohio State University Wexner Medical CenterColumbusOhioUnited States; 3 Division of Surgical Oncology 12306 The Ohio State University Wexner Medical CenterColumbusOhioUnited States

## A case presentation



**Video 1** Endoscopic-ultrasound guided gastrojejunostomy creation to relieve afferent limb syndrome in a post-Whipple patient using an electrocautery-enhanced lumen apposing metal stent system, resulting in effective decompression and drainage.


**Fig. 1:**
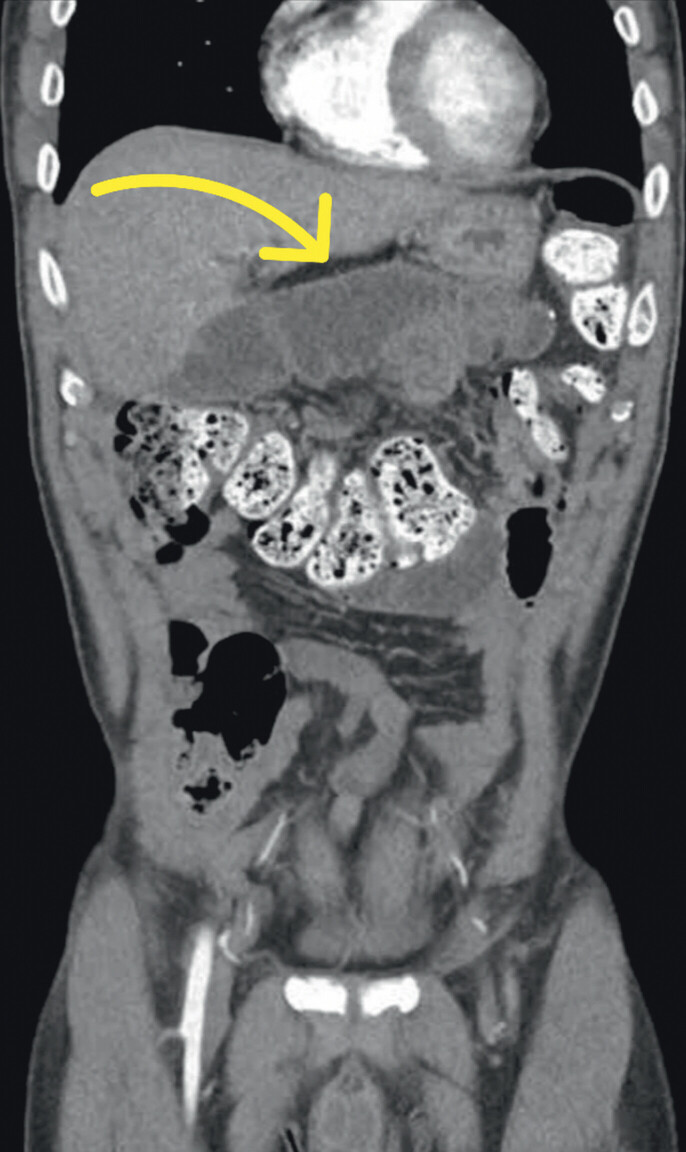
An index CT scan showing the dilated afferent limb, highlighted by the yellow arrow.

Afferent limb syndrome is an uncommon mechanical complication following surgically altered gastrointestinal anatomy. Its incidence can range from 0.3 to 1.0% in most series, but has been reported up to 13% after the Whipple procedure. 1​ 2​ ​3​ We report a 54‑year‑old man with early-stage pancreatic adenocarcinoma status after the remote Whipple procedure who presented with 1 week of progressive epigastric pain, persistent vomiting, and inability to tolerate oral intake. Computed tomography (CT) demonstrated a moderately dilated afferent limb consistent with closed‑loop obstruction ( Fig. 1) . An exploratory laparotomy was attempted, but dense adhesions and recurrent tumor caused the small bowel and colon to be firmly adherent to the mass, making bypass or resection unsafe due to high enterotomy risk. Surgery was aborted, and endoscopic management pursued. Endoscopy revealed a moderately dilated limb and a severe extrinsic stenosis about 40 cm from the anastomosis, which was traversed using gentle pressure, suction, and water immersion ( Video 1) .

**Fig. 2:**
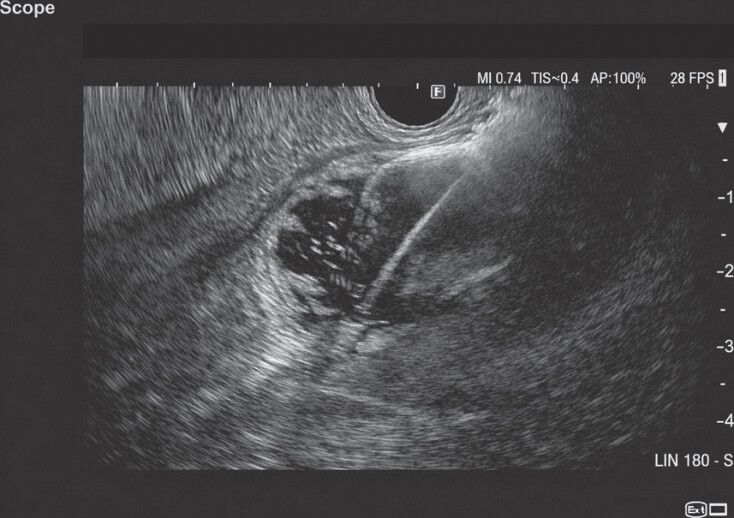
An endosonographic view of LAMS stent deployment with electrocautery-enhanced tip to create GJ anastomosis.

Since the patient had a prognosis beyond 6 months and luminal stenting, direct jejunostomy, and percutaneous transhepatic drainage were not feasible, the decision was made to create a novel gastrojejunostomy (GJ) anastomosis using an electrocautery‑enhanced lumen‑apposing metal stent (LAMS). Given the marked dilation beyond the stricture, a decompression tube was placed to distend the lumen with sterile water and a contrast mixture for targeted endoscopic ultrasound-guided GJ. After confirming an optimal gastric site without interposed vasculature, the electrocautery‑enhanced LAMS system was advanced, and a 20×10 mm stent was deployed with both flanges securely apposed ( **Figs.** 2 **and** 3) . Repeat CT scan and immediate drainage of a contrast-mixture and air entry into the jejunum confirmed patency and effective decompression ( Fig. 4) . The patient was eventually transitioned to a clear liquid diet and weaned off intravenous pain medications. He was able to resume a regular diet with normalization of liver function tests and was discharged 48 hours later.

**Fig. 3:**
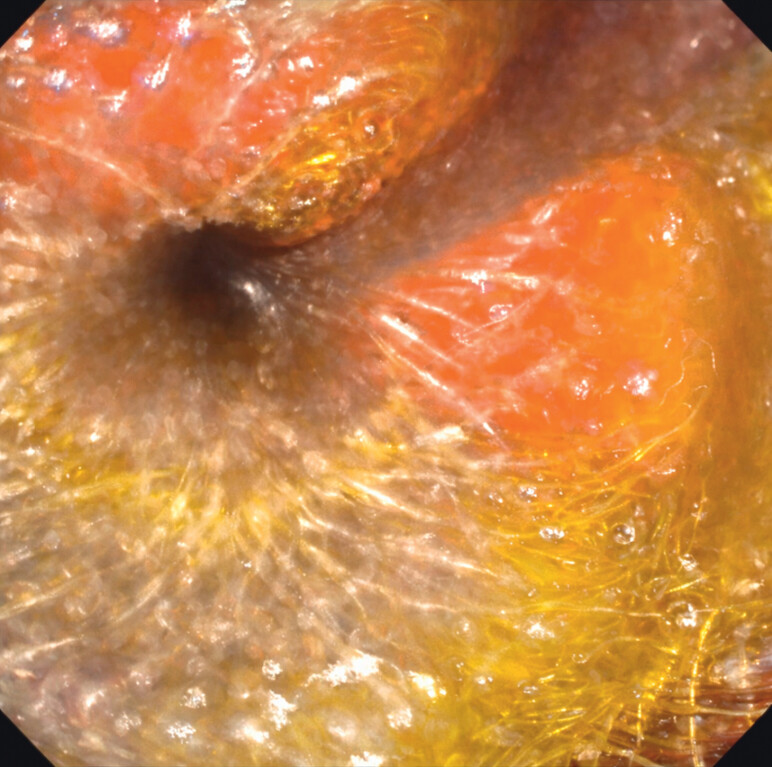
An endoscopic view of LAMS deployment after the creation of a novel GJ anastomosis.

**Fig. 4:**
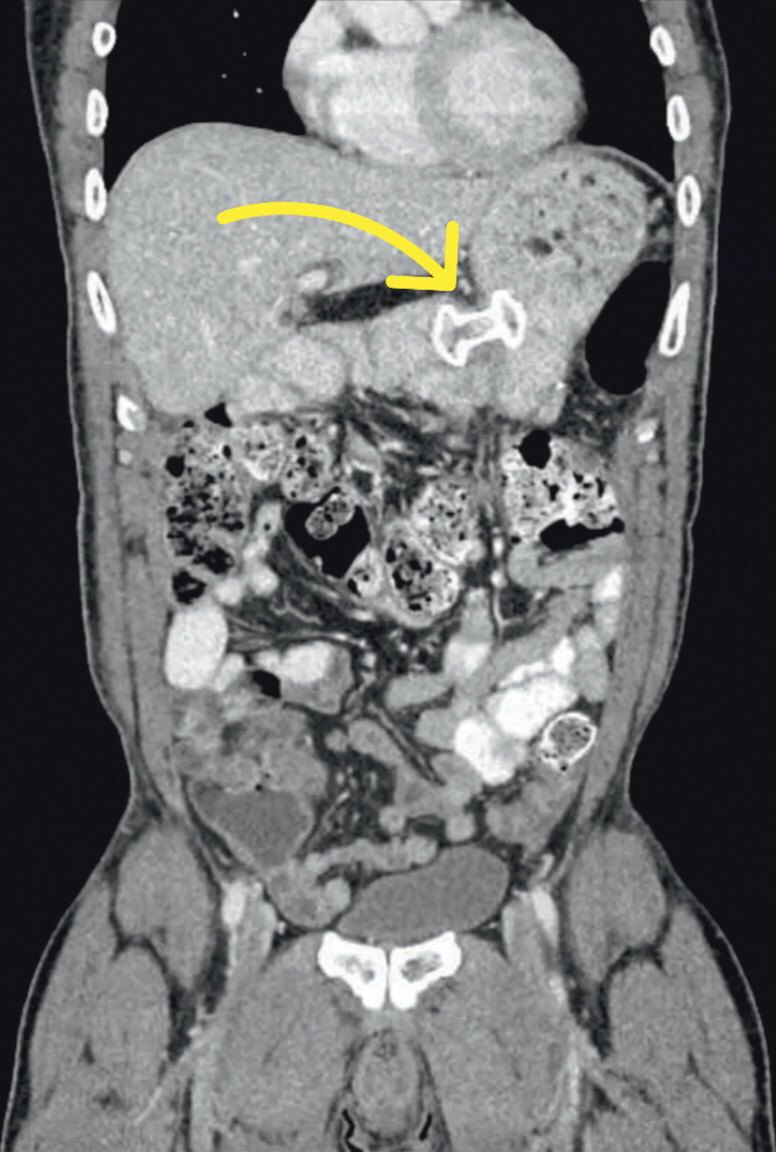
Post-LAMS deployment CT scan showing decreased dilation of the afferent limb with the LAMS in place, highlighted by the yellow arrow.

Endoscopy_UCTN_Code_TTT_1AS_2AK
